# Dosimetric Study Comparing 3D Conformal Radiotherapy (3D-CRT), Intensity Modulated Radiotherapy (IMRT) and Volumetric Modulated Arc Therapy (VMAT) in Hypofractionated One-Week Radiotherapy Regimen in Breast Cancer

**DOI:** 10.7759/cureus.31860

**Published:** 2022-11-24

**Authors:** Aftab Ahmad, Saikat Das, Vipin Kharade, Manish Gupta, V.P. Pandey, Anju K.V., Ilamparithi Balasubramanian, Rajesh K Pasricha

**Affiliations:** 1 Department of Radiation Oncology, All India Institute of Medical Sciences, Bhopal, Bhopal, IND

**Keywords:** breast radiotherapy, vmat, imrt, fast forward, hypofractionated radiotherapy

## Abstract

Introduction

Recently, the one-week hypofractionated radiotherapy regimen (26 Gy in 5 fractions) for adjuvant breast radiotherapy has been shown to be non-inferior to other hypofractionated regimens (15-16 fractions). The aim of the present dosimetric study is to compare Intensity Modulated Radiotherapy (IMRT), Volumetric Modulated Arc Therapy (VMAT) and 3D Conformal Radiotherapy (3D-CRT) for a one-week hypofractionated radiotherapy regimen (26 Gy in 5 fractions) for adjuvant breast radiotherapy.

Methods

A total of 30 patients with histologically proven invasive carcinoma of the breast after breast conservation surgery (BCS) or modified radical mastectomy (MRM) were considered for *in silico* planning study. The dose prescription used was 26 Gy in 5 fractions as used in the FAST Forward protocol. Targets were contoured according to standard guidelines. The heart, ipsilateral lung, and contralateral breast were contoured as organs at risk.

Results

Planning Target Volume (PTV) coverage: For IMRT, VMAT and 3D-CRT, respectively, the volumes that received at least 95% of the prescription dose (V_95_) were 95.7 ± 2.12, 92.47 ± 3.83, 90.87 ± 5.13; mean PTV doses (D_mean_) were 26.1 ± 0.6, 25.7 ± 0.7, and 28 ± 4.39 (3D-CRT has higher D_mean_ compared to other techniques). Maximum PTV doses (D_max_) were 28.23 ± 0.72, 28.73 ± 0.64, and 29.8 ± 1.03. IMRT had a better V_95_ coverage and conformity index.

Organs At Risk (OARs): The volumes that received at least 25% of the prescription dose (V_25_) of the heart were 3.41 ± 4.7, 1.8 ± 2.02 and 4.3 ± 6.98 in IMRT, VMAT and 3D-CRT, respectively. The volumetric (V_25_) comparison of heart dose in left-sided breast cancer was significantly different between VMAT and 3D-CRT (p=0.04, Wilcoxon signed-rank test). The volume that received at least 5% of the prescription dose (V_5 _)_ _was less than 25% in the 3D-CRT plan (12.55). For the ipsilateral lung, the V_25_ parameters were 19.53 ± 10.96, 23.93 ± 13.58 and 20.5 ± 12.32 in IMRT, VMAT and 3D-CRT, respectively.

Conclusion

From this study, we can conclude that IMRT and VMAT techniques are feasible and can achieve better dosimetric goals for target and OARs though minimizing the area achieving low dose remains to be a dosimetric concern for VMAT.

## Introduction

Optimal radiotherapy fractionation in breast cancer has been a matter of debate for many decades. A better understanding of the radiosensitivity of breast tissue (α/β ratio) has supported the use of moderate hypofractionation using 15-16 fractions [1]. This, however, does not represent the limit of this approach, and recently, the one-week adjuvant radiotherapy trial (FAST-Forward) has been reported to be safe, effective, and non-inferior alternative, and potentially practice changing [2]. The FAST-Forward trial showed that post-operative 26 Gy in 5 fractions of 5·2 Gy is non-inferior to 40 Gy in 15 fractions over 3 weeks within an absolute 1·6% non-inferiority margin in terms of local recurrence [2]. In the FAST-Forward trial, 3D conformal radiation therapy (3D-CRT) with tangential field arrangements was used. It was suggested in the trial to seek the guidance of the quality assurance (QA) team in the case of inverse planning with tighter margins [2]. Though the one-week regimen is cost effective, with a lesser burden to the machine, achieving the organ at risk (OAR) dose constraints remains challenging [3]. It is imperative that for use of such a hypofractionated regimen strict dosimetric quality assurance is needed. Recently, in view of several logistic advantages of Intensity Modulated Radiotherapy (IMRT)/Volumetric Modulated Arc Therapy (VMAT), the dosimetric comparison between different techniques is clinically relevant. The aim of this comparative *in silico* study was to compare the dosimetric parameters of 26 Gy in 5 fractions, as used in the FAST-Forward trial, between 3D-CRT, IMRT and VMAT techniques to assess the feasibility and challenges.

## Materials and methods

This is a prospective, single-centre, non-randomized, observational *in silico* dosimetric study, to compare IMRT, VMAT and 3D-CRT for 26 Gy in a 5-fraction regimen. This study was done over a period of 14 months from December 2020 to January 2022. The study was approved by the Institutional Human and Ethical Committee (IHEC) of All India Institute of Medical Sciences Bhopal, Bhopal, India.

Sample size

CT- simulation imaging data of 30 patients (aged between 18-70 years) diagnosed with breast cancer planned for radiotherapy was used to generate IMRT, VMAT and 3D-CRT plans. The sample size was tailored as per the distribution of the variables (non-parametric/Poisson’s distribution) keeping the logistic issues, patient inflow, availability and the COVID-19 pandemic situation during the period of the study under consideration.

Simulation

Patients were positioned on breast board and a thermoplastic ray cast was used to aid in immobilization and reproducibility of position throughout imaging and treatment. Patients were instructed to lie supine with their heads tilted away from the affected side and arms abducted to elevate them above the head. In this position, the patient-specific immobilization device was prepared and labelled with identifiers. The mid-axillary fold (ipsilateral), post-operative breast or chest wall scars, boundaries of palpable breast tissue and drain sites (if present) were all marked with radio-opaque markers if needed. Treatment planning and repositioning were aided by the use of external fiducial markers. Free-breathing CT images were taken on Optima CT-580 RT (GE Healthcare, India), 3 mm slice thickness from the angle of the jaw to the lower border of the L2 vertebra. CT scan images acquired were transferred to the radiation therapy contouring and planning workstation, MONACO treatment planning system (TPS) (version 5.11.02, Elekta, Stockholm, Sweden). Target volume and OAR, i.e., heart, ipsilateral and contralateral lung and contralateral breast were delineated as per the guidelines [2].

IMRT planning

For IMRT planning, seven coplanar intensity modulated fields were used. The couch and collimator were kept at zero degrees. The gantry angle ranged between 145-330. The IMRT plans were optimized to cover at least 95% of the planned target volume (PTV) with 95% of the prescribed dose while reducing the exposure to OARs to the greatest extent possible. Monaco TPS was used to make inverse planning optimizations (version 5.11.02). The dose was calculated using the Monte Carlo technique for IMRT and VMAT and collapsed cone convolution algorithm for 3D-CRT with a grid resolution of 3 mm. To expand the fluence outside the body contour, an auto flash margin of 1.5 cm to PTV was applied. Tissue inhomogeneities were taken into account during the treatment planning optimization procedure. The optimization process was used with as many iterations as were required to meet the planning objectives.

VMAT planning

Continuous gantry motion was modelled as a series of discrete angle segments, and multi-leaf collimators (MLC) with 5 mm width apertures were progressively added throughout optimization in Monaco TPS (version 5.51.10). A variable dosage rate was used to maximize monitor unit (MU)per gantry angle. The incremental angle of the gantry was preserved at one degree. With heterogeneity corrections and a 3 mm grid size, the Monte Carlo dose calculation algorithm was utilized. To expand the fluence outside the body contour, an auto flash margin of 1.5cm to PTV was applied.

3D-CRT planning

3D-CRT planning was made using a mono iso-centric technique. The chest wall was irradiated using two tangential beams. Gantry angles ranged from 300° to 335° for the medial fields and from 130° to 155° for the lateral fields. The dose was prescribed to the isocenter. Field‑in‑field technique with MLCs was used to provide adequate coverage of the PTV (95% of the prescribed dose to 95% of the target volume) and to reduce the hot spots to <110%. For all plans, 6 MV photons with a dose rate of 300 MU were used. The treatment planning system used collapsed cone convolution algorithm for 3D-CRT with a grid resolution of 3 mm.

Plan evaluation

Dose-volume histograms (DVH) were used to evaluate all plans found satisfactory with respect to PTV coverage and avoidance of OAR according to the protocol criteria. DVHs were used for evaluation and comparisons of dose to OARs and all the parameters required for the study. Standard dose constraints for OARs were followed [4].

Dose Volume Histogram (DVH) Parameters

The conformity index (CI) describes the degree to which the prescribed isodose volume conforms to the shape and size of the target volume.

CI = V^2^_Rx_/TV*V_RI_

TV = Structure volume, VRx = structure volume covered by the dose of interest, V_RI_ = total volume of dose of interest.

The homogeneity index (HI) describes the uniformity of dose within a target volume and is directly calculated from the statistics of the DVH of the MONACO planning software.

HI = D_5_%/D_95_%

Statistical analysis

This is a non-randomized prospective study and the principal aim of this project was to find agreement trajectories between dosimetric comparison, sample size was tailored as per the distribution of variables (non-parametric/Poisson’s distribution). In keeping with the logistic issues, patient inflow and COVID-19 situation, a sample size of 30 was achieved. The mean and SD of the dose volume parameters were calculated and analysis of variance (ANOVA) was used for comparing the mean in all cases. p<0.05 was considered statistically significant.

## Results

Patient- and tumor-related characteristics

A total of 30 patients were included in the study. There were 17 patients with left-sided and 13 patients with right-sided breast cancer. The mean age was 42.26 (range: 29-70) years. Adjuvant radiotherapy was planned for eight patients after breast conservation surgery (BCS) and 22 patients in Post-Mastectomy Radiotherapy (PMRT) setting.

Planning Target Volume (PTV) coverage

The mean Planning Target Volume (PTV) volume was 962.7 cc. The PTV coverage for IMRT, VMAT and 3D-CRT techniques for dosimetric comparison is depicted in Table 1. The mean dose (D_mean_) was 26.1 ± 0.6, 25.7 ± 0.7, and 28 ± 4.39 for IMRT, VMAT and 3D-CRT, respectively (3D-CRT has higher D_mean_ compared to the other techniques). The maximum dose (D_max_) was 28.23 ± 0.72, 28.73 ± 0.64, and 29.8 ± 1.03 for IMRT, VMAT and 3D-CRT, respectively. V_95_ ± SD were 95.7 ± 2.12, 92.47 ± 3.83, and 90.87 ± 5.13 for IMRT, VMAT and 3D-CRT, respectively (IMRT has better V_95_ coverage). The homogeneity index (HI) was significantly different for IMRT and VMAT (vs 3D-CRT). CI was significantly higher for IMRT compared to 3D-CRT. The conformity index was higher in 3D-CRT than in VMAT.

**Table 1:** Comparison of dosimetric parameters of PTV coverage PTV: Planning Target Volume; IMRT: Intensity Modulated Radiotherapy; VMAT: Volumetric Modulated Arc Therapy; 3D-CRT: 3D Conformal Radiotherapy; V_95_: Volume that received at least 95% of the prescription dose; D_mean_: Mean PTV dose; D_max_: Maximum PTV dose; HI: Homogenity Index; CI: Conformity Index

	IMRT	VMAT	3D-CRT	IMRT vs VMAT (p-value)	IMRT vs 3D-CRT (p-value)	VMAT vs 3D-CRT (p-value)
D_mean (Gy)_	26.1 ± 0.6	25.77 ±0.72	28 ± 4.39	0.98	0.000	0.000
D_Max (Gy)_	28.23 ±0.72	28.73 ±0.64	29.8 ±1.03	0.000	0.002	0.18
V_95 (%)_	95.7 ±2.12	92.47 ±3.83	90.87 ±5.13	0.76	0.06	0.002
HI	1.09 ±0.04	1.11 ±0.39	1.22 ±0.12	0.98	0.000	0.000
CI	0.66 ±0.09	0.41 ±0.16	0.52 ±0.13	0.14	0.000	0.03

Comparison in BCS and PMRT setting

In this study, eight patients received radiotherapy in the BCS setting and 22 patients in the PMRT setting. Table 2 shows the dosimetric comparison of different techniques and comparative p-values. D_max_ of 3D-CRT was higher; 29.47 ± 0.65 and 29.93±0.99 for BCS and modified radical mastectomy (MRM) (p<0.05). The V_95_ and CI of IMRT plans were higher (95.99± 0.82, CI: 0.71 and 95.25±2.87, CI: 0.66 for BCS and PMRT, respectively).

**Table 2:** Comparison of dosimetric parameters of PTV coverage (BCS and MRM) PTV: Planning Target Volume; IMRT: Intensity Modulated Radiotherapy; VMAT: Volumetric Modulated Arc Therapy; 3D-CRT: 3D Conformal Radiotherapy; V_95_: Volume that received at least 95% of the prescription dose; D_mean_: Mean PTV dose; D_max_: Maximum PTV dose; HI: Homogenity Index; CI: Conformity Index

BCS (n=8)						
Technique	IMRT	VMAT	3D-CRT	P value		
				IMRT vs VMAT	IMRT vs 3D-CRT	VMAT vs 3D-CRT
D_mean (Gy)_	26.39± 0.22	25.90± 0.57	26.53± 0.73	0.25	0.89	0.07
D_Max (Gy)_	28.74± 0.38	28.49± 0.52	29.47± 0.65	0.65	0.05	0.003
V_95 (%)_	95.99± 0.82	95.43± 2.24	93.3±3.89	0.91	0.20	0.27
HI	1.09±0.01	1.09± 0.03	1.24± 0.15	1.00	0.01	0.00
CI	0.71± 0.05	0.53± 0.21	0.54± 0.13	0.11	0.15	0.99
MRM (n=22)						
D_mean (Gy)_	26.22±0.71	25.73±0.59	28.50±5.08	0.87	0.07	0.004
D_Max (Gy)_	28.35±0.86	28.50±0.58	29.93±0.99	0.83	0.000	0.000
V_95 (%)_	95.25±2.87	92.71±3.86	90.03±5.26	0.15	0.002	0.06
HI	1.10±0.05	1.10±0.04	1.21±0.10	0.97	0.000	0.000
CI	0.66±0.11	0.43±0.15	0.51±.14	0.000	0.011	0.10

Dosimetric evaluation of organs at risk

Table 3 shows that the V_25_ of the heart was 3.41 ± 4.7, 1.8 ± 2.02 and 4.3 ± 6.98 in IMRT, VMAT and 3D-CRT, respectively. The volumetric (V_25_) comparison of heart dose in left-sided breast cancer was significantly different between VMAT and 3D-CRT (p=0.04, Wilcoxon signed-rank test). V_5_ was less than 25% (12.55) in 3D-CRT plan. For the ipsilateral lung, the V_25_ parameter was 19.53 ± 10.96, 23.93 ± 13.58 and 20.5 ± 12.32 in IMRT, VMAT and 3D-CRT, respectively.

**Table 3:** Dosimetric parameters of organs at risk IMRT: Intensity Modulated Radiotherapy; VMAT: Volumetric Modulated Arc Therapy; 3D-CRT: 3D Conformal Radiotherapy;  D_mean_: Mean dose to contralateral breast;  V_25_: Volume that received at least 25% of the prescription dose

Organ at risk	Parameter	IMRT	VMAT	3D-CRT
Heart	V_25_	3.41 ± 4.7	1.8 ± 2.02	4.3 ± 6.98
Heart (for left-sided tumor)	V_25_	7.29 ±0.53	2.8 ±0.19	7.6 ±0.78
Ipsilateral Lung	V_25_	19.53 ± 10.96	23.93 ± 13.58	20.5 ±12.32
Contralateral breast	D_mean_	2.10 ± 0.96	3.47 ± 0.77	1.30± 0.65

## Discussion

This study is an *in silico *comparison of IMRT, 3D-CRT and VMAT planning for 1 week of hypofractionated adjuvant radiotherapy in breast cancer. The addition of radiotherapy reduces locoregional recurrence rate and improves survival in breast cancer and the one-week regimen has been shown to be feasible and effective [1]. Our study shows all three techniques are feasible, though IMRT and 3D-CRT have an overall better dosimetric outcome, similar to that reported in the literature [4].

In radiotherapy treatment, the therapeutic goal is to achieve higher tumor control probability (TCP) and lower the normal tissue complication probability (NTCP). The fractionation regimens of radiotherapy used in breast cancer have evolved beyond conventional fractionation to regimens involving significant dose hypofractionation. Keeping these two factors in mind, dosimetric evaluation of the planning technique is imperative to achieve a higher therapeutic ratio (TCP/NTCP). The 26 Gy in 5 fractions regimen has been shown to be safe and effective; this study compares the feasibility of different planning techniques for this regimen.

The target volume of the chest wall poses a challenge in contouring due to significant variation. Respiratory movement adds to the level of uncertainties. As the lateral limit of the chest wall field is determined by latissimus dorsi which can extend posterior to the mid-axillary line, adequate coverage of the chest wall using tangential beams often include a significant volume of lung and cardiac tissue in the left side [5]. Our study showed V_25_ was lower in VMAT plans particularly in left-sided breast cancer but 3D-CRT was superior for low dose parameter (V_5_) which is consistent with the literature [4].

In a study, Ma et al. reported that IMRT has a superior dosimetric profile compared to 3D-CRT and VMAT though the latter has better PTV coverage [6]. The same was reported by Sudha et al, who found PTV coverage was better in VMAT [5]. A study by Das Majumdar et al. showed improved PTV coverage with VMAT and IMRT compared to 3D-CRT [7]. In a study by Liu et al., IMRT has shown better V_95_ coverage compared to VMAT (p-value <0.05) [8]. Even though the dose conformity is better with VMAT compared to 3D-CRT, the possible limitations of VMAT could be the sharp fall off of dose and strict adherence to the dosimetric recipe (95% dose to PTV). It has higher coverage of normal organs with a lower dose. The conformity index for the hypofractionated regimen used in our study was better for IMRT compared to VMAT or 3D-CRT (p<0.05). A similar result was reported in previous studies for other hypofractionated regimens [5-7]. In the multicentric randomized trial protocol (HYPORT-Adjuvant) treatment planning achieving breast/chest wall coverage with 95% of the prescribed dose and limiting the maximum dose to less than 107% of the prescribed dose was allowed [9].

There is considerable variation in dosimetric constraints to compare hypofractionated schedules. An attempt was made to adhere to the specified constraints of the OAR to the extent possible without compromising PTV coverage. In view of fewer comparative dosimetric studies for the one-week regimen, the pattern was compared with more protracted hypofractionated regimens. The V_25_ value for lung reported by Das Majumdar et al. were 13.46, 13.15, and 12.12 for 3D-CRT, IMRT and VMAT, respectively, with no statistically significant difference [7]. V_20_ Gy reported by Sudha et al. were 29.88 and 12.79 for 3D-CRT and VMAT, respectively [5]. In the one-week radiotherapy regimen (26 Gy in 5 fractions), lung constraints (V_30_<17%) were achieved in 30% of patients by the 3D-CRT technique [3]. VMAT or IMRT techniques can be explored to improve coverage. Radiation-induced heart disease has complex pathophysiology and is postulated to be related to microvascular occlusion. Several dosimetric studies have investigated the dosimetric implications of breast radiation on cardiac risk, especially for left-sided tumor and internal mammary chain radiation [10,11]. A study done by Garg and Kumar has shown that V_25_ to the heart with 3D-CRT was 14.13 and IMRT 27.74 Gy [12]. In a dosimetric study comparing post-mastectomy IMRT and 3D-CRT, Khullar et al. showed that except for the mean dose to the opposite breast, the compliance to DVH constraints for PTV and OARs was significantly improved with IMRT [13]. A systematic review and meta-analysis by Taylor et al. showed that in left-sided breast cancer, the mean heart dose averaged over 149 studies from 28 countries was 5.4 Gy [11]. Liu et al. reported D_mean _± SD dose to the contralateral breast to be 6.25, 6.67 and 8.44 for 3D-CRT, IMRT and VMAT, respectively, concluding mean dose was more in VMAT as compared to other techniques [14]. It supports our result where the mean dose was higher in VMAT as compared with other techniques, and in this aspect, 3D-CRT achieved a lower dose to the contralateral breast [8].

The possibility of using an arc-based IMRT approach with a standard MLC leads to significantly reduced treatment time while improving dose conformity at the expense of volume of low dose coverage. This is achieved in VMAT by simultaneously changing the position of the MLCs, the dose rate, and the gantry speed during patient treatment. In a prospective study of 153 breast conservation and mastectomy patients, Munshi et al. observed that short tangential arcs in VMAT planning for breast and chest wall irradiation resulted in breast dose compatible with reduced dose to heart and lung [15]. Comparative dosimetric studies have shown VMAT as a feasible method for post-mastectomy radiotherapy [14]. There is however a paucity of studies comparing newer techniques with 3D-CRT in the one-week adjuvant radiotherapy regimen in breast cancer [4]. The geometric uncertainties during treatment (systematic and random errors) may lead to a variation in the dose of PTV. This underlines the importance of quality assurance during the treatment execution of an extremely hypofractionated one-weekly regimen. This problem is postulated to be lower in 3D-CRT compared to VMAT, but achieving adequate coverage of PTV remains a challenge in 3D-CRT and often at the cost of overdosing to the OAR [5].

Investigations into IMRT and VMAT techniques have been explored in nodal irradiation which was not required in the majority of patients in the early hypofractionation regimen trials. Fogliata et al. conducted a dosimetric study with highly restrictive planning objectives in order to limit the low-dose irradiation with maximum possible target dose homogeneity, and observed that methods like deep inspirational breath-hold technique are used for heart dose reduction [16]. A study done by Kivanc et al. comparing five different techniques for chest wall and lymphatic irradiation in patients with left‐sided breast cancer found that IMRT was better than 3D-CRT in several dosimetric parameters [17]. Rastogi et al., comparing dose distribution of IMRT and 3D-CRT on the left-sided chest wall of breast cancer patients with post-mastectomy, concluded that IMRT has better conformity of plan. It also reduces the mean dose of ipsilateral lung and heart as compared to 3D-CRT in high-dose volume, but 3D-CRT is superior in terms of low-dose volume [18].

In a study by Piras et al., a dosimetric comparison of VMAT and 3D-CRT for 26 Gy in 5 fractions showed 3D-CRT better for V_5_ (11.89% vs 29.47%) compared to VMAT even though the conformity index was higher in VMAT (0.89 vs. 0.627 in 3D-CRT). Reduction in undesirable doses to the OAR can be of advantage especially when most of the patients are exposed to systemic treatment including chemotherapy. The biological significance of low-dose radiation exposure needs further exploration [4]. 

The use of an ultra hypofractionated one-week radiotherapy regimen for adjuvant breast radiotherapy will require critical analysis of the risk-benefit ratio, especially regarding late toxicity. A study by Krug et al. showed that relative risks of significant normal tissue effects for patients treated with 26 Gy increased significantly (p<0.001) over time for all normal tissue effects (NTE) except for telangiectasia compared to patients treated with 40 Gy [19].

Nevertheless, the present study has several limitations. In this study, we did not use deep inspiration breath holding (DIBH) or tracking methods in planning or treatment. Further studies with larger sample sizes and stratified sampling (whole breast/chest wall radiation or left/right-sided tumor) are needed for further improvement of results.

## Conclusions

Hypo-fractionated radiotherapy has been established as an acceptable treatment regimen for adjuvant radiotherapy in breast cancer in view of non-inferiority to conventional fractionation. This clinical experience and evidence are attributed to the radiobiological characteristic of breast cancer tissue and the results of various randomized trials and meta-analyses. In the newer hypo-fractionation regimen shrinking the overall treatment time to one week, all three techniques are feasible though each one has its advantages and limitations. Our study shows that IMRT has better target coverage (V_95_) and conformity index. For left-sided tumors, a significantly lower V_25_ value for the heart was achieved with the VMAT technique though the V_5_ parameter was superior with 3D-CRT. The dose to heart for a right-sided tumor was achieved within the dose constraints and the dose to the contralateral breast was minimized with the 3D-CRT technique for right-sided tumors. From this study, we can conclude that IMRT and VMAT techniques can achieve better dosimetric goals for target and ipsilateral OARs, though minimizing the area achieving a low dose remains a dosimetric concern for VMAT.
